# ECG-gated CT improves diagnosis in prosthetic valve degeneration

**DOI:** 10.1093/ehjimp/qyae049

**Published:** 2024-05-21

**Authors:** Jonathan X Fang, Tiberio M Frisoli, Gennaro Giustino, Pedro A Villablanca, Pedro Engel Gonzalez, Brian P O’Neill, Dee Dee Wang, William W O’Neill, James C Lee

**Affiliations:** Center for Structural Heart Disease, Henry Ford Health System, 2799 West Grand Boulevard, Detroit, MI 48202, USA; Center for Structural Heart Disease, Henry Ford Health System, 2799 West Grand Boulevard, Detroit, MI 48202, USA; Center for Structural Heart Disease, Henry Ford Health System, 2799 West Grand Boulevard, Detroit, MI 48202, USA; Center for Structural Heart Disease, Henry Ford Health System, 2799 West Grand Boulevard, Detroit, MI 48202, USA; Center for Structural Heart Disease, Henry Ford Health System, 2799 West Grand Boulevard, Detroit, MI 48202, USA; Center for Structural Heart Disease, Henry Ford Health System, 2799 West Grand Boulevard, Detroit, MI 48202, USA; Center for Structural Heart Disease, Henry Ford Health System, 2799 West Grand Boulevard, Detroit, MI 48202, USA; Center for Structural Heart Disease, Henry Ford Health System, 2799 West Grand Boulevard, Detroit, MI 48202, USA; Center for Structural Heart Disease, Henry Ford Health System, 2799 West Grand Boulevard, Detroit, MI 48202, USA

**Keywords:** echocardiogram, computed tomography, aortic valve replacement

An 81-year-old woman with a prior valve-in-valve transcatheter implantation (TAVI) of a 23 mm CoreValve (Medtronic, USA) inside a 21 mm Carpentier (Edwards Lifesciences, USA) bioprosthesis 9 years ago and coronary artery bypass grafting 20 years ago presented dyspnoea and slow-rising pulse with an aortic area ejection-systolic murmur on physical examination. Transthoracic echocardiogram showed a left-ventricular ejection fraction of 59%, aortic transvalvular peak/mean gradients of 6.7/4.1 mmHg, aortic valve area (AVA) of 1.91 cm^2^, and no aortic regurgitation (*Figure 1A*). The valve was not well-visualized on parasternal view due to acoustic shadowing from the frame, and all standard did not reveal any significant gradient. Owing to discrepancy between clinical and echocardiographic finding, cardiac catheterization was pursued, which showed patent grafts but an aortic peak/mean gradient of 110/69 mmHg with an AVA of 0.37 cm^2^ (*Figure 1B*). Heart team evaluation for suspected bioprosthetic valve degeneration had computed tomography (CT) done as part of the workup (*Figure 1C*), showing a vertical-take-off aortic accentuated by the CoreValve, with predicted coaxial alignment of the valve achievable from a left subcostal view (*Figure 1D*). Transthoracic echocardiogram from this off-axis view showed aortic transvalvular peak/mean gradients of 116/81 mmHg, AVA of 0.28 cm^2^ (*Figure 1E*). The patient had prohibitive surgical risk and underwent a valve-in-valve TAVI with a 20 mm Sapien 3 valve (Edwards Lifesciences, USA) (*Figure 1F*) per heart-team decision. This case demonstrates the importance of multimodality approach integrating clinical, imaging, and catheterization findings in diagnosing bioprosthetic degeneration when each modality alone has limitation.

**Figure 1:**
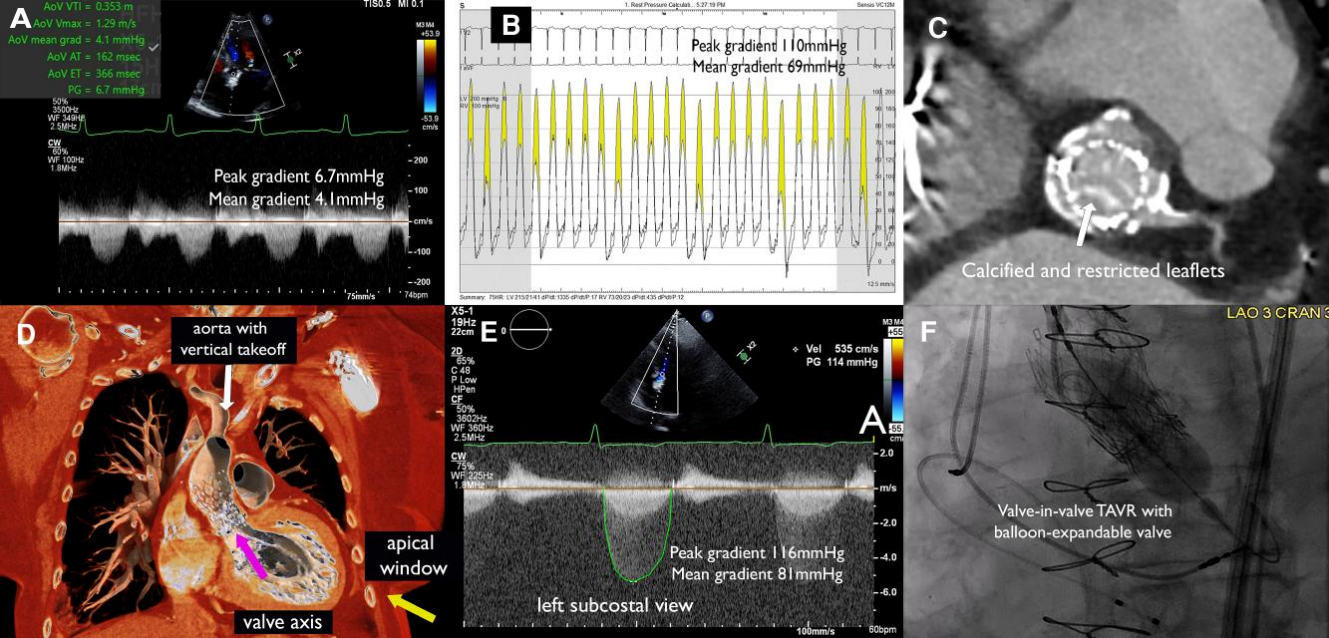


## Consent

Patient consent has been obtained for educational use of material, including publication.

## Data Availability

No new data were generated or analysed in support of this research.

## Lead author biography

Jonathan X. Fang, MBBS, is currently the structural heart disease fellow at Henry Ford Hospital, Detroit, MI, USA. He obtained his medical degree and completed his training in internal medicine and cardiology at the University of Hong Kong. He then pursued further training in the USA including an Interventional Cardiology Fellow at Brigham and Women’s Hospital, Boston, USA, and his current structural heart disease fellowship. He has published articles in journals such as JACC: Cardiovascular intervention, Catheterization & Cardiovascular Interventions, and Heart Rhythm.

